# Multiscale Modeling of Hospital Length of Stay for Successive SARS-CoV-2 Variants: A Multi-State Forecasting Framework

**DOI:** 10.3390/v17070953

**Published:** 2025-07-06

**Authors:** Minchan Choi, Jungeun Kim, Heesung Kim, Ruarai J. Tobin, Sunmi Lee

**Affiliations:** 1 Department of Applied Mathematics, Kyung Hee University, Yongin 17104, Republic of Korea; choi7186@khu.ac.kr; 2 Department of Mathematics and Computer Science, Korea Science Academy of KAIST, Busan 47162, Republic of Korea; jkim.math@gmail.com; 3 Department of Internal Medicine, College of Medicine, Chungbuk National University Hospital, Chungbuk National University, Cheongju 28644, Republic of Korea; clint74@gmail.com; 4 Melbourne School of Population and Global Health, The University of Melbourne, Melbourne, VIC 3010, Australia; ruarai.tobin@unimelb.edu.au

**Keywords:** COVID-19, hospital length of stay, South Korea, multi-state model, parameter estimation

## Abstract

Understanding how hospital length of stay (LoS) evolves with successive SARS-CoV-2 variants is central to the multiscale modeling and forecasting of COVID-19 and other respiratory virus dynamics. Using records from 1249 COVID-19 patients admitted to Chungbuk National University Hospital (2021–2023), we quantified LoS across three distinct variant phases (Pre-Delta, Delta, and Omicron) and three age groups (0–39, 40–64, and 65+ years). A gamma-distributed multi-state model—capturing transitions between semi-critical and critical wards—incorporated variant phase and age as log-linear covariates. Parameters were estimated via maximum likelihood with 95% confidence intervals derived from bootstrap resampling, and Monte Carlo iterations yielded detailed LoS distributions. Omicron-phase stays were 5–8 days, shorter than the 10–14 days observed in earlier phases, reflecting improved treatment protocols and reduced virulence. Younger adults typically stayed 3–5 days, whereas older cohorts required 8–12 days, with prolonged admissions (over 30 days) clustering in the oldest group. These time-dependent transition probabilities can be integrated with real-time bed-availability alert systems, highlighting the need for variant-specific ward/ICU resource planning and underscoring the importance of targeted management for elderly patients during current and future pandemics.

## 1. Introduction

Since its emergence, COVID-19 has imposed sustained pressure on health systems worldwide, with hospital occupancy closely tracking each dominant SARS-CoV-2 variant [1,2]. In South Korea, an adaptive social-distancing tier initially stipulated hospitalization for all confirmed cases, regardless of disease severity. However, the explosive spread of the Delta variant in mid-2021 and the even larger Omicron wave at the end of 2021 quickly saturated available beds, forcing a strategic pivot to home isolation for patients with mild or moderate symptoms while reserving hospital resources for the most severe cases [3]. These policy shifts highlight that incidence alone is an incomplete predictor of surge capacity demands: the duration of hospitalization (LoS) significantly influences ward and ICU turnover. Timely and accurate estimates of LoS are pivotal for the multiscale modeling and forecasting of COVID-19 and other respiratory viruses, as they inform bed-capacity planning and help healthcare managers anticipate staffing requirements [4,5,6,7]. In general, LoS distributions tend to be heavily right-skewed, particularly among older adults or immunocompromised patients. These distributions may also vary substantially with changing SARS-CoV-2 variants due to factors such as viral virulence, therapeutic advancements, and vaccination status. Omitting age-stratified or variant-specific effects can lead to underestimation of peak hospital demand and exacerbate patient flow bottlenecks, especially in higher-risk groups.

Methodological approaches for analyzing LoS span a broad spectrum, reflecting the complexity of hospital admission dynamics. Mixture regression and finite-mixture cure models have been used to capture multiple subpopulations or “cure” processes [8,9,10], while multi-state frameworks can account for transitions across different care levels [11,12,13]. Competing risk regression for discharge versus death has proven useful for understanding clinical endpoints [14], whereas agent-based or compartmental models can provide system-level perspectives on how LoS patterns affect epidemiological trajectories [15,16]. Recent research indicates that the Delta variant elongated median LoS, while Omicron markedly shortened it [17,18], pointing to the importance of continuous monitoring as new variants emerge. However, many studies lack a probabilistic, multiscale perspective that can seamlessly update transition probabilities in response to shifting epidemic conditions.

For South Korea specifically, existing work either reports LoS findings from a limited time window [19,20] or explores individual predictors of prolonged admissions [21]. Yet, no published study has simultaneously integrated ward progression (e.g., non-critical to semi-critical to critical care), age stratification, and variant-phase effects into a unified framework. Without such a comprehensive approach, hospital planners cannot readily translate epidemic forecasts into actionable bed-capacity estimates that account for heterogeneous patient pathways. Furthermore, the interplay of individual-level factors (e.g., age and comorbidities) with population-level factors (e.g., variant prevalence) underscores the need for multiscale models to ensure robust forecasting and resource allocation.

To address these gaps, we propose a Bayesian model and data-fusion approach that combines patient-level length-of-stay observations with a gamma-distributed competing-risk multi-state model. We incorporate variant phase (Pre-Delta, Delta, Omicron) and age group as log-linear covariates on transition rates, thereby capturing both within-ward dynamics and potential escalations to higher-care settings. Uncertainty is quantified through bootstrap resampling and 10,000 Monte Carlo draws, enabling a more complete representation of the plausible range of patient outcomes. Specifically, our objectives are to

1. Derive phase-specific LoS distributions and quantify how age modifies these durations;
2. Estimate time-dependent probabilities for transitions to critical care, discharge, or death, providing a direct interface for bed-availability alert systems;
3. Assess parameter identifiability and stability given a moderate sample size;
4. Demonstrate that variant- and age-adaptive estimations of LoS parameters yield accurate projections of ward- and ICU-specific demand in different epidemic contexts.

By linking infection and hospitalization dynamics in a single modeling framework, our work offers an evidence-based foundation for prioritizing hospital resources, designing dynamic patient flow protocols, and managing surge capacity during both current and future SARS-CoV-2 waves. More broadly, our approach aligns with ongoing efforts in the multiscale modeling and forecasting of respiratory viruses, illustrating how data-driven updates to patient transition rates can significantly improve the accuracy and timeliness of pandemic response measures.

## 2. Data and Methods

### 2.1. Data

**Figure 1:**
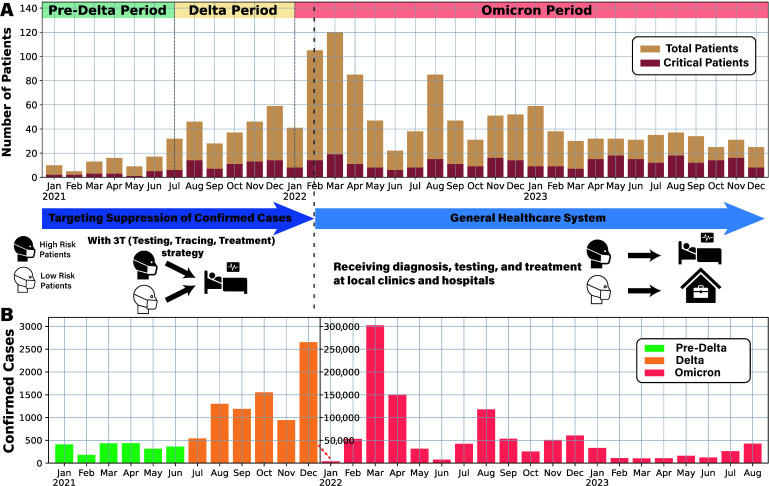
Overview of patient admissions and severity analysis at Chungbuk National University Hospital. (**A**) Monthly data of patients admitted from 2021 to 2023 are presented, with yellow-hued bar graphs indicating total patient counts and red-hued bar graphs representing critical cases. (**B**) Confirmed COVID-19 cases in Chungbuk regions from January 2021 to August 2023 are depicted. The graph was constructed using regional COVID-19 data from the Korea Disease Control and Prevention Agency, and because Korea transitioned from comprehensive to sample-based surveillance following the reclassification to a level 4 infectious disease on 31 August 2023, only data up to August 2023 were available for plotting. Due to the surge in cases during the Omicron period, the graph scale was adjusted mid-way, marked by a red dashed line and a new axis.

After applying all preprocessing filters, including removal of records with missing age, duplicates, and unknown vital status, we retained 1483 COVID-19 patients admitted to Chungbuk National University Hospital from 1 January 2021 to 31 December 2023. The curated dataset contains, for each patient, a unique identifier, age, survival outcome, admission and discharge dates, and the complete sequence of ward transfers (ward numbers and transfer dates). Since all patients were admitted to a university hospital, they were classified as severe cases despite variations in disease severity. The timeline in Figure 1A shows the monthly changes in hospitalized and critically ill patients at the hospital, while Figure 1B illustrates the overall trend in COVID-19 incidence in Chungbuk Province. Although confirmed cases surged with the dominance of the Omicron variant, the number of hospital admissions increased only modestly due to the transition to the General Healthcare System, indicating a shift in resource allocation strategies.

The length of hospital stay (LoS) was a central focus of our study, and to assess patient outcomes effectively, we applied three key classification criteria. First, we categorized patients based on their date of admission, recognizing that the emergence of dominant variants has been shown to affect both disease incidence and the length of hospital stay. Specifically, we defined the three variant phases as follows: Pre-Delta from 1 January to 31 July 2021, Delta from 1 August 2021 to 15 January 2022, and Omicron from 16 January 2022 onward, based on the timelines of dominant variant circulation reported in prior studies [22,23]. Second, we divided patients into three age groups (0–39 years, 40–64 years, and 65+ years), given that age is a crucial factor in influencing LoS and healthcare resource utilization [24]. Finally, clinical severity was also considered a critical factor directly linked to LoS, as patients with higher severity levels typically experience longer stays and require more intensive care [25]. This classification allowed us to explore how LoS varied across different epidemic phases, age groups, and disease severity levels, providing valuable insights into hospital resource management and treatment efficiency.

### 2.2. Gamma Distribution

Understanding the temporal and age-related variations in hospital length of stay (LoS) is critical for optimizing healthcare resource management, especially during evolving epidemics. To analyze these variations, we performed gamma cumulative distribution fitting on LoS data to estimate transition probabilities during hospitalization, providing insights into patient flow between hospital wards and overall system dynamics. Details regarding the choice of distribution are provided in Supplementary Materials, where we compare the gamma distribution with Weibull and log-normal alternatives using Akaike Information Criterion (AIC) scores. To capture the temporal effects of different epidemic waves, we categorized LoS data into three distinct epidemic phases (Pre-Delta, Delta, and Omicron). By fitting the gamma cumulative distribution to each phase, we assessed how changes in viral transmissibility, disease severity, and healthcare policies influenced hospitalization patterns. This phase-specific analysis is essential for understanding how healthcare demands shift over time and for adapting hospital strategies accordingly.

In addition to temporal trends, we examined the age-dependent characteristics of LoS by classifying patients into three age groups: 0–39, 40–64, and 65+ years. By fitting the gamma cumulative distribution separately for each group, we quantified differences in LoS and transition probabilities, revealing significant variations in hospitalization duration across age groups. Given that older patients typically require longer hospital stays and more intensive care, this age-based analysis helps inform resource allocation and capacity planning. By integrating both temporal and age-specific perspectives, our study provides a more comprehensive understanding of hospitalization dynamics, supporting evidence-based decision-making for hospital management and policy development. The gamma cumulative distribution function used for this analysis is defined as follows [26,27]:

(1) $$F_{k}\left(x;\alpha_{k},\beta_{k}\right)=\int_{0}^{t}\frac{1}{\beta_{k}^{\alpha_{k}}\Gamma \left(\alpha_{k}\right)}t^{\alpha_{k}-1}e^{-\frac{t}{\beta }}dt$$

where *x* represents the length of stay, $\alpha_{k}$ is the shape parameter, and $\beta_{k}$ is the scale parameter. The cumulative distribution function (CDF) parameters for the gamma fitting were estimated using the gamma function and curve fitting techniques available in the stats and optimization modules of the scipy package in Python 3.9 [28,29,30]. This approach allowed us to accurately model hospital flow patterns and optimize resource allocation based on length of stay and transition dynamics.

### 2.3. Probabilistic Transition Model

**Figure 2:**
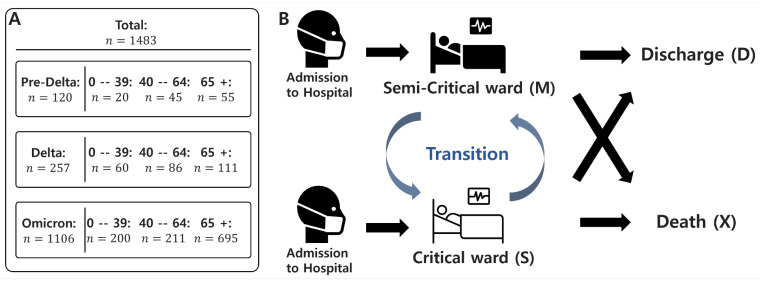
Data Summary and model diagram of patient state transitions. (**A**) Data summary: A total of 1483 patient records are included in the analysis. For each of the three epidemic periods (Pre-Delta, Delta, and Omicron), the number of patients is stratified by age group (0–39, 40–64, and 65+). Detailed counts are provided for each subgroup to support the model calibration. (**B**) The diagram illustrates the patient state transitions in the model. Patients are admitted in either a semi-critical or critical state, and transitions between these two wards are allowed during hospitalization. However, once a patient transitions to a death or discharge state, no further transitions occur.

We develop a probabilistic transition model based on the competing risks framework, extending it into a Markov model to analyze patient state transitions and length of stay (LoS) during the COVID-19 pandemic [31]. Our model assumes a time-homogeneous Markov process, where the probability of transitioning between states depends only on the current state and not on the duration of time spent in state or prior transitions. Figure 2A presents the classification and distribution of the dataset used in the model, while Figure 2B illustrates the overall model framework. The model tracks the evolution of patient states over time, calculating both transition and survival probabilities on an episode-by-episode basis. The model defines key patient states encountered during hospitalization:

- Upon admission, a patient is placed in either a semi-critical ward (M) or a critical ward (C).
- If the patient recovers, they transition to the discharged state (D), which is an absorbing state with no further transitions.
- If the patient’s condition deteriorates to death, they transition to the death state (X), another absorbing state.

State transitions occur within the competing risks framework, meaning that once a patient enters an absorbing state (D or X), no further transitions take place [32]. To account for variations in hospitalization patterns, we define the following:

- Epidemic waves: Pre-Delta, Delta, and Omicron, denoted as *w*.
- Age groups: 0–39, 40–64, and 65+, denoted as *a*.
- State transitions: Represented as *k*.

Each transition *k* is modeled using a gamma distribution, with the probability density function at time *t* given by the following [26,27]:

(2) $$f_{k}\left(t;\alpha_{k},\beta_{k}\right)=\frac{1}{\beta_{k}^{\alpha_{k}}\Gamma \left(\alpha_{k}\right)}t^{\alpha_{k}-1}e^{-\frac{t}{\beta }}$$

The daily transition functions are interpreted as conditional on the transition path realized, as defined under the competing risks approach. Parameters $\alpha_{k}$ and $\beta_{k}$ incorporate the effects of the infection wave (*w*) and patient age (*a*) through the following log-linear model.

(3) $$\begin{array}{c} \log\alpha_{k}\left(w,a\right)=\mu_{\alpha_{k}}+\gamma_{\alpha_{k}}\left(w\right)+\delta_{\alpha_{k}}\left(a\right)\\ \log\beta_{k}\left(w,a\right)=\mu_{\beta_{k}}+\gamma_{\beta_{k}}\left(w\right)+\delta_{\beta_{k}}\left(a\right) \end{array}$$

Here, $\mu_{\alpha_{k}}$ and $\mu_{\beta_{k}}$ represent the log-scale baseline shape parameter and scale parameter for transition *k*. $\gamma_{\alpha_{k}}\left(w\right)$ and $\gamma_{\beta_{k}}\left(w\right)$ add the effect of infection wave *w*, while $\delta_{\alpha_{k}}\left(a\right)$ and $\delta_{\beta_{k}}\left(a\right)$ add the effect of age *a*. These terms enter additively on the log-scale; they appear in the gamma parameters as the multiplicative factors. That is, in this model, the parameters $\alpha_{k}$ and $\beta_{k}$ of the gamma distribution are log-linearly scaled by *w* and *a*. Within each episode, the transition probability $f_{k}\left(t\right)$ and the survival probability (for m≠k) for the competing transition $S_{m}\left(t\right)=1-\int f_{m}\left(t\right)dt$ are computed. Thus, the probability that a particular transition *k* will occur is $f_{k}\left(t\right)\Pi_{m\neq k}S_{m}\left(t\right)$. The model follows the Markov property, meaning that state transitions at each time step depend solely on the current state. For each patient episode, we compute transition and survival probabilities at every time step, generating a time-specific log-likelihood value. The overall likelihood function is then obtained by summing the log-likelihoods across all *N* episodes, as given below:

(4) $$L\left(\theta \right)=\sum_{i=1}^{N}\left[\log f_{k}\left(t_{i};\alpha_{k}\left(w_{i},a_{i}\right),\beta_{k}\left(w_{i},a_{i}\right)\right)+\sum_{m\neq k}\log S_{m}(t_{i};\alpha_{m}\left(w_{i},a_{i}\right),\beta_{m}\left(w_{i},a_{i}\right)\right]$$

where *i* represents each episode and θ represents the entire set of model parameters. Maximum likelihood estimation (MLE) is performed by maximizing this overall likelihood function to obtain the optimal parameter estimates $\hat{\theta }$ [32,33]. We conducted simulations to reconstruct state transition paths for each episode by calculating transition probabilities using the maximum likelihood estimate (MLE) parameters, $\hat{\theta }$. Each simulation was repeated 10,000 times, enabling a quantitative assessment of the hospital length of stay (LoS) distribution and the computation of the mean final LoS for patients. The initial admission ward was assigned probabilistically, with probabilities derived directly from the data and detailed in Supplementary Materials. Additionally, to evaluate the reliability of the estimated LoS, we applied the bootstrap method to compute 95% confidence intervals [34].

## 3. Results

### 3.1. Data Analysis

**Figure 3:**
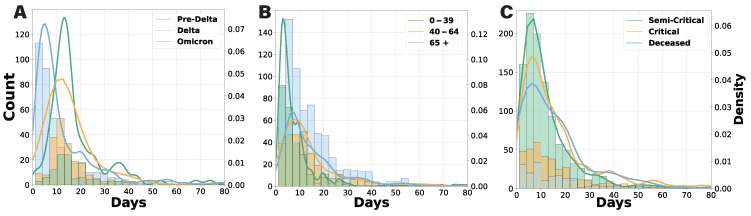
Histogram and KDE plots of total hospital length of stay by various grouping criteria. (**A**) Results by admission date, comparing the Pre-Delta, Delta, and Omicron periods. (**B**) Results by age group (0–39, 40–64, and 65+ years). (**C**) Results by clinical severity (semi-critical, critical, and deceased), with any patient who visited a critical care unit classified as critical.

In this section, we analyze the distribution of hospital stays and transition probabilities across different variant periods, age groups, and severity levels. Figure 3 presents histograms and kernel density estimation (KDE) plots illustrating the length of hospital stays based on these factors. The KDE method provides smooth probability density estimates, facilitating a clearer visualization of distribution patterns. Patients were categorized by three key criteria: epidemic phase (determined by the dominant variant), age group, and clinical severity. Our analysis indicates that hospital stays during the Pre-Delta and Delta phases were predominantly between 10 and 14 days, with a more concentrated distribution in the Pre-Delta phase—likely due to stricter hospitalization policies and a lower overall patient count. In contrast, during the Omicron phase, the average hospital stay shortened to 5 to 8 days, suggesting a milder disease course and revised hospitalization guidelines.

Age-specific analysis revealed a significantly higher number of hospitalized patients aged 65 and older. Patients aged 0 to 39 typically stayed for 3 to 5 days, while those aged 40 to 64 and 65+ had longer stays of 8 to 12 days. Notably, hospitalizations exceeding 30 days were primarily observed in the 65+ group. In terms of clinical severity, patients discharged after a critical admission were classified as “critical”. While the maximum hospital stay ranged from 7 to 10 days across all severity groups, the overall length of stay distribution widened from semi-critical to critical and deceased patients. The critical cases exhibited the greatest variability, highlighting the unpredictable clinical course of COVID-19 in critically ill individuals.

### 3.2. Daily Transition Probability Analysis

**Figure 4:**
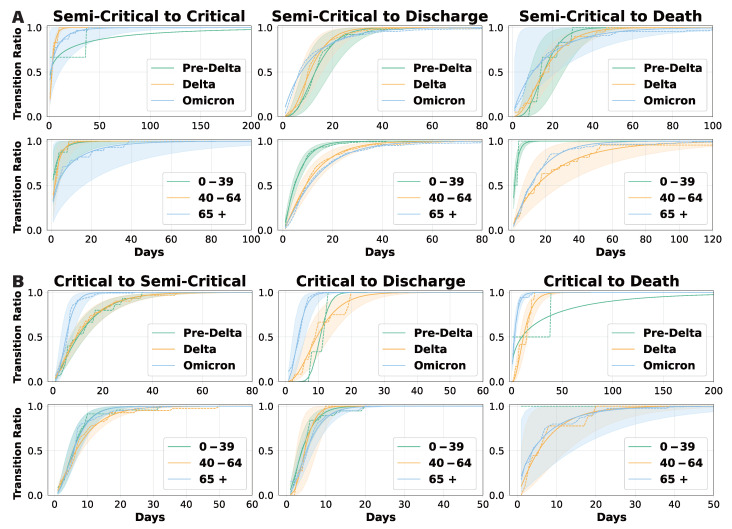
Cumulative daily transition probabilities based on gamma fitting. Conditional cumulative transition probabilities under the competing risks framework. That is, for each transition type and stratified group, the curve shows the cumulative probability that the patient completes that specific transition by a given day, assuming that the competing transitions do not occur. (**A**) shows the transitions from the semi-critical ward, and (**B**) shows the transitions from the critical ward.

We conducted a gamma fitting analysis on ward length-of-stay data to derive cumulative daily transition functions (Figure 4). Each cumulative probability curve represents the conditional probability that a transition of interest occurs by a given day, conditional on that transition being the outcome for the patient. For example, the semi-critical-to-death curve indicates the probability that death occurs directly from the semi-critical ward within a specific number of days, assuming this is the terminal state reached. Since the cumulative probability reaches 1 when all patients have completed a transition, a lower curve indicates a longer transition duration. For transitions with sparse data, such as those from critical to death in the 0–39 age group, only the empirical data are displayed. In Figure 4A, the transition from the semi-critical to critical ward reveals a distinct pattern: patients during the Pre-Delta period experienced significantly longer transitions than those in later phases, and patients aged 65+ transitioned notably slower than those in the 0–39 and 40–64 age groups. In contrast, the cumulative probability curves for transitions from semi-critical care to discharge or death remained relatively consistent across epidemic phases, though younger patients (0–39) completed these transitions more quickly. In addition, the somewhat counterintuitive transition from semi-critical to death was observed in 136 patients, which is a larger number than initially expected. Among them, 112 were elderly, and the time to transition tended to increase with age. This finding suggests that direct deterioration from a semi-critical state without escalation to intensive care may reflect either clinical constraints or age-related differences in care pathways and prognosis.

Figure 4B illustrates transition curves for patients in the critical ward, including those returning to a semi-critical ward, being discharged, or passing away. These curves show minimal variation across age groups, suggesting that age has a limited effect on transition times in critical care. However, transitions during the Omicron period were significantly shorter, indicating a faster disease course or changes in clinical management. Overall, our findings suggest that patient age primarily influences transition durations in semi-critical care, whereas transitions from the critical ward are more dependent on the epidemic phase and viral strain. These insights have important clinical implications: older semi-critical patients may require closer monitoring, while the accelerated transitions observed during the Omicron period highlight the need for adaptable resource allocation and treatment strategies. Detailed gamma distribution parameters can be found in Supplementary Materials.

### 3.3. Model-Based Analysis of Hospital Length of Stay

**Figure 5:**
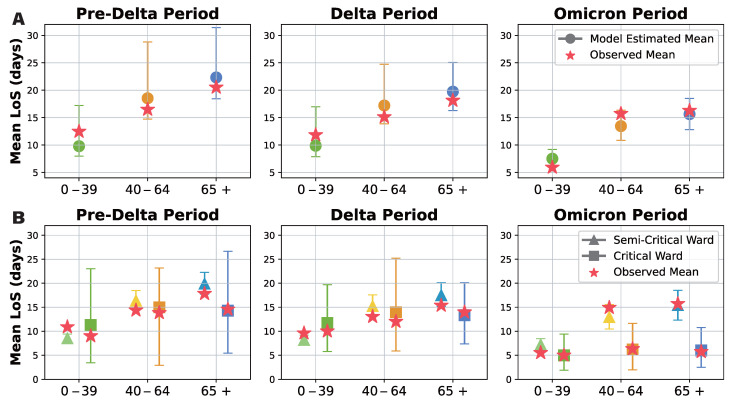
Probabilistic transition model results for hospital length of stay analysis. Green, orange, and blue indicate results for ages 0–39, 40–64, and 65+. Circles, triangles, and squares show average stays overall, in semi critical wards, and in critical wards. Stars show actual data averages for comparison. (**A**) Overall hospital length of stay model estimates for each dominant variant period (Pre-Delta, Delta, Omicron) subdivided by age group (0–39, 40–64, 65+). Observed means marked with stars; model estimates with 95% confidence intervals marked with circles. (**B**) Mean durations in the semi-critical and critical wards for each dominant variant period subdivided by age group (0–39, 40–64, 65+). Semi-critical durations indicated by triangles; critical durations indicated by squares; observed means shown as stars; model estimates with 95% confidence intervals provided.

Our probabilistic transition model was developed to address the limitations of previous studies, which were unable to analyze the effects of epidemic phase and age independently due to data constraints. A summary of our model results is presented in Figure 5A, with detailed findings available in Supplementary Materials. While the mean estimates remain relatively consistent across all transitions, the confidence intervals progressively narrow from the Pre-Delta to Delta and Omicron periods, indicating that model estimates become more robust as data accumulate. Additionally, total hospital length of stay increases with patient age but gradually decreases from the Pre-Delta to Omicron periods.

To further examine differences in hospitalization duration, we compared the mean length of stay in semi-intensive and intensive care units across different age groups, considering only patients with a non-zero length of stay (Figure 5B). The estimated mean length of stay was generally comparable between groups. However, confidence intervals for intensive care unit stays were notably wider than those for semi-intensive care, reflecting greater variability and lower certainty in these estimates. Nevertheless, as more data were collected over time, the confidence in these estimates improved. Notably, our results confirm that patients tend to stay longer in semi-intensive care than in intensive care, and that hospitalization duration increases with age. While length of stay in intensive care units remained relatively consistent across age groups during the Omicron period, semi-intensive care unit stays increased significantly with age, further differentiating the two ward types.

Our probabilistic transition model provided a more detailed and reliable analysis of the impact of epidemic phase and age on patient outcomes, overcoming the limitations of gamma fitting in sparse data scenarios. The results reveal that while overall hospital length of stay follows a similar trend, confidence in the estimates improves as data accumulate. Additionally, hospital stays increase with age but decrease over successive epidemic phases. The prolonged stays observed in semi-intensive care units, particularly among older patients, underscore the need for age- and phase-specific clinical management strategies and optimized healthcare resource allocation.

## 4. Discussion

We investigated the length of hospital stay (LoS) and ward transfer dynamics of COVID-19 patients admitted to Chungbuk National University Hospital from 1 January 2021 to 31 December 2023, within the broader scope of the multiscale modeling and forecasting of COVID-19 and respiratory virus dynamics [15,35]. By leveraging detailed hospitalization data across three distinct epidemic phases (Pre-Delta, Delta, and Omicron) and stratifying by patient age (0–39, 40–64, and 65+ years), we reconstructed a granular view of disease progression. Specifically, our gamma-distributed Markov competing-risks framework captured transitions between semi-critical and critical care, along with discharge or death, revealing how evolving policies, changing viral characteristics, and age-related risks collectively shape hospital utilization.

Our findings confirm that LoS patterns and ward transfer pathways are markedly influenced by epidemic stage and patient age. During the Pre-Delta period, mandatory hospitalization policies [36] drove prolonged LoS, reflecting early cautious management strategies aimed at containing viral spread. In contrast, Delta-phase admissions showed systematically shorter stays, likely due to more refined clinical protocols and relaxed hospitalization criteria. The Omicron phase further reduced LoS, aligning with observations of lower virulence and improved treatments [37]. Patients in critical care often experienced shorter total LoS, possibly stemming from rapid clinical deterioration, early mortality, or an expedited decision-making process in ICU settings [38,39,40,41].

These observations underscore the importance of integrating time-varying admission and transition patterns into multiscale models for respiratory viruses [42]. Effective hospital resource allocation relies on the accurate, dynamic forecasting of patient flow, where epidemic conditions, viral characteristics, and patient demographics interact in a nonlinear fashion [16]. In this study, the probabilistic Markov framework allowed us to accommodate right-skewed LoS distributions and partial data limitations [12,31]. By incorporating epidemic phase and age through log-linear covariates in a gamma distribution model [43], we derived ward-specific LoS estimates and inter-ward transition rates. Such detailed modeling can feed directly into real-time dashboards or bed-management systems, facilitating timely alerting and scenario planning as conditions evolve [44].

As more data were gathered over successive phases, we observed that bootstrap-derived confidence intervals narrowed, reinforcing the reliability of our approach. This real-time learning capability aligns well with the goals of multiscale modeling, wherein local patient data inform immediate clinical decisions, and broader system-level inferences feed back into public health policies. Crucially, our findings indicate that ignoring phase-specific LoS patterns and age-based risks could lead to the systematic underestimation or overestimation of hospital capacity requirements, with significant implications for ICU overflow and resource bottlenecks.

Despite offering valuable insights, our study has several limitations. First, we modeled patient trajectories using a time-homogeneous Markov framework, which necessarily omitted patient-level covariates—such as comorbidities, immunity or vaccination status, antiviral therapy, and socioeconomic factors. This simplification also excludes non-Markovian, history-dependent dynamics that can arise during prolonged or severe hospitalizations and influence both outcomes and length of stay. Future, more detailed models should explicitly capture these effects by using history-augumented models. Second, because our dataset comes from a single institution, the generalizability of our findings to other hospitals and geographic contexts may be limited; future research should, therefore, collect and analyze data from multiple institutions to validate these results across diverse care environments. Third, while gamma-based parameterization captured key features of the LoS distributions, it could not simultaneously account for extensive stratification by both age and epidemic phase. Future iterations might apply higher-dimensional modeling strategies or hierarchical approaches to reduce the reliance on separate phase or age stratifications. Lastly, the relatively wide confidence intervals observed in critical care durations highlight the need for larger, multicenter datasets to refine estimates and bolster their predictive utility.

Our results underscore the critical role of data-driven modeling in guiding resource allocation, especially for an ever-evolving pathogen like SARS-CoV-2. Future work should explore the feasibility of integrating additional clinical indicators into the competing-risks framework, potentially employing multi-state or agent-based models that account for stochastic, individual-level variations. Moreover, our probabilistic outputs for LoS can be merged with larger, system-level simulators that capture regional transmission dynamics, thereby uniting hospital-based observations with population-wide forecasts. A key advance would be to systematically incorporate time-dependent vaccination coverage, antiviral therapy availability, and real-time sequencing data for emerging variants.

By systematically analyzing variations in hospitalization patterns across epidemic phases and age strata, our model provides a critical evidence base for policymakers, hospital administrators, and public health officials. Built on gamma-distributed multi-state transitions and log-linear covariates, the modeling framework itself is broadly applicable to other acute care contexts beyond COVID-19. For instance, the same structure could be used to forecast hospital demand during influenza surges, RSV outbreaks, or future respiratory pandemics that exhibit age-specific severity profiles and dynamic care transitions. In future research, applying our approach to multicenter datasets and incorporating additional covariates—such as comorbidity profiles—will enhance the precision and utility of forecasting tools for COVID-19 and other respiratory pathogens. Ultimately, robust, adaptive modeling frameworks are essential for effective outbreak mitigation, enabling healthcare systems to anticipate capacity constraints, guide triage protocols, and refine interventions as new variants emerge.

## Data Availability

The original contributions presented in this study are included in the article/Supplementary Materials. Further inquiries can be directed to the corresponding author.

## Supplementary Materials

The following supporting information can be downloaded at: https://www.mdpi.com/article/10.3390/v17070953/s1. Supplementary S1. Data-Derived Initial Admission Ward Probabilities; Supplementary S2. Selection of Distribution; Supplementary S3. Gamma Distribution Parameter Estimation; Supplementary S4. Model Estimation Result.
