# Supplementary material for: Multiscale Modeling of Hospital Length of Stay for Successive SARS-CoV-2 Variants: A Multi-State Forecasting Framework
Source: Viruses. 2025 Jul 6;17(7):953. doi: 10.3390/v17070953 (PMC12299293; doi:10.3390/v17070953)
Supplement: Supplementary file 1 [file viruses-17-00953-s001.zip › Supplementary_file_S1.pdf]

Supplementary S1. Data-Derived Initial Admission Ward Probabilities

Table S1-1.

Probabilities used in the simulation model to determine each patient's initial hospitalization status (Semi-Critical / Critical). The probabilities, derived from the data for each age group across the Pre-Delta, Delta, and Omicron periods, are used to assign the initial state for each patient within the simulation.

Period	Age	Semi-Critical	Critical
**Pre-Delta period**	**0 – 39**	0.88	0.12
	**40 – 64**	0.85	0.15
	**65 +**	0.80	0.20
**Delta period**	**0 – 39**	0.86	0.14
	**40 – 64**	0.82	0.18
	**65 +**	0.75	0.25
**Omicron period**	**0 – 39**	0.86	0.14
	**40 – 64**	0.82	0.18
	**65 +**	0.75	0.25
