# Supplementary material for: Multiscale Modeling of Hospital Length of Stay for Successive SARS-CoV-2 Variants: A Multi-State Forecasting Framework
Source: Viruses. 2025 Jul 6;17(7):953. doi: 10.3390/v17070953 (PMC12299293; doi:10.3390/v17070953)
Supplement: Supplementary file 1 [file viruses-17-00953-s001.zip › Supplementary_file_S2.pdf]

## Supplementary S2. Selection of Distribution

To assess the suitability of the gamma distribution used in the model, we compared its fit against log-normal and Weibull distributions. For model comparison, we employed the Akaike Information Criterion (AIC), which combines the maximized log-likelihood and the model's degrees of freedom (i.e., the number of estimated parameters), and is defined as:

$$AIC = -2\ln\hat{L} + 2k$$

Where $\hat{L}$ is the maximum likelihood and $k$ is the number of parameters. The term $-2\ln\hat{L}$ serves as an estimate proportional to the Kullback–Leibler divergence between the true data-generating distribution and the candidate model, while the additional penalty term $2k$ discourages overfitting by accounting for model complexity. AIC is thus designed to select the model that minimizes information loss. In contexts such as modeling hospital length of stay, where likelihood-based fitting is feasible and candidate distributions differ in parameter complexity, AIC offers a theoretically grounded and practically useful criterion.

**Table S2-1.**

**Comparison of AIC score between Gamma / Weibull / Lognormal distribution.** The table presents a comparison of AIC scores based on fitting the length-of-stay data for each ward using three candidate distributions—gamma, Weibull, and log-normal.

| Transition | Gamma | Weibull | Lognormal |
|---|---|---|---|
| **Semi-Critical to Critical** | 261.9164 | 259.9464 | 247.0933 |
| **Semi-Critical to Discharge** | 7071.8777 | 7106.3652 | 6930.0726 |
| **Semi-Critical to Death** | 1089.0659 | 1085.5083 | 1061.5249 |
| **Critical to Semi-Critical** | 1121.5484 | 1136.5462 | 1108.9775 |
| **Critical to Discharge** | 359.3442 | 362.6009 | 362.0190 |
| **Critical to Death** | 301.1163 | 301.7588 | 295.1592 |

Although the gamma distribution did not consistently yield the lowest AIC across all transition types, it exhibited either superior or practically equivalent fit in most cases. Among the six transitions evaluated (‘Semi-Critical to Critical’, ‘Semi-Critical to Discharge’, ‘Semi-Critical to Death’, ‘Critical to Semi-Critical’, ‘Critical to Discharge’, and ‘Critical to Death’), the gamma distribution achieved the best AIC or showed a modest but meaningful improvement over the Weibull and log-normal alternatives for transitions such as ‘Semi-Critical to Discharge’ or ‘Critical to Discharge’. In transitions like ‘Critical to Death’ and ‘Semi-Critical to Critical’, the AIC differences between gamma and Weibull were minimal, indicating comparable model fit. While the log-normal distribution yielded better AIC scores for ‘Semi-Critical to Death’ and ‘Critical to Death’, these advantages likely stem from sensitivity to a small number of extreme values in the distribution tail.

Overall, all three candidate distributions—gamma, Weibull, and log-normal—demonstrated sufficient robustness and plausibility, and our use of the gamma distribution aligns with prior studies in hospital length-of-stay modeling. Employing a single distribution across all transitions further promotes consistency in interpretation and mathematical tractability, particularly for tail behavior and sojourn time estimation. Based on these considerations, we adopted the gamma distribution for the current analysis. Nonetheless, given the stable performance of all three distributions, future researchers may reasonably select among them according to their data characteristics and modeling needs.
