# Supplementary material for: Multiscale Modeling of Hospital Length of Stay for Successive SARS-CoV-2 Variants: A Multi-State Forecasting Framework
Source: Viruses. 2025 Jul 6;17(7):953. doi: 10.3390/v17070953 (PMC12299293; doi:10.3390/v17070953)
Supplement: Supplementary file 1 [file viruses-17-00953-s001.zip › Supplementary_file_S3.pdf]

Supplementary S3. Gamma Distribution Parameter Estimation

Table S3-1.

Gamma Fitting Parameter Estimates for Ward Transitions (from Semi-Critical ward). This table presents the shape and scale estimates (with confidence intervals) for transitions from the Semi-Critical to the Critical ward, to discharge, or death, across six groups (Pre-Delta, Delta, Omicron, 0–39, 40–64, 65+). Two rows per group show the shape (upper) and scale (lower) parameters. Transitions lacking sufficient data for gamma fitting (where only cumulative probabilities were available) are omitted.

		Semi-Critical to Critical	Semi-Critical to Discharge	Semi-Critical to Death
Pre-Delta period	Shape	0.1667 (0.0184, 0.4723)	3.8422 (2.9198, 4.8394)	4.8763 (3.3045, 6.5052)
	Scale	139.7063 (1.5618, 273.4172)	3.6939 (3.0940, 4.2888)	3.4168 (2.7184, 4.1468)
Delta period	Shape	1.2900 (1.1483, 1.4303)	3.2269 (2.5007, 3.9450)	2.4986 (2.025, 3.0677)
	Scale	2.6932 (2.3222, 3.0812)	3.7221 (3.0938, 4.3493)	7.1140 (6.2450, 7.9844)
Omicron period	Shape	0.3474 (0.1302, 0.5412)	0.8793 (0.2177, 1.6018)	0.8117 (0.0595, 3.4128)
	Scale	16.8442 (1.5659, 39.5880)	12.6342 (1.6405, 17.2055)	19.5934 (1.7866, 26.9060)
0 – 39	Shape	0.3677 (0.2683, 0.5252)	1.3820 (1.2421, 1.5233)	1.0348 (0.8776, 1.2039)
	Scale	5.7386 (1.5399, 14.6154)	4.4206 (3.8822, 4.9655)	2.0859 (1.5765, 2.5838)
40 – 64	Shape	0.8857 (0.6386, 1.2372)	1.3482 (1.1651, 1.5548)	0.7951 (0.6171, 0.9986)
	Scale	3.0070 (1.4660, 4.3302)	8.8320 (7.7120, 9.9509)	29.5461 (1.5111, 41.6760)
65 +	Shape	0.4033 (0.1047, 0.6583)	1.2702 (0.1526, 5.5911)	1.1760 (0.1417, 5.9965)
	Scale	19.6519 (2.5368, 43.4730)	10.9729 (1.5988, 13.3596)	12.7388 (1.6234, 16.2988)

Table S3-2.

Gamma Fitting Parameter Estimates for Ward Transitions (from Critical ward). This table presents the shape and scale estimates (with confidence intervals) for transitions from the Critical to the Semi-Critical ward, to discharge, or death, across six groups (Pre-Delta, Delta, Omicron, 0–39, 40–64, 65+). Two rows per group show the shape (upper) and scale (lower) parameters. Transitions lacking sufficient data for gamma fitting (where only cumulative probabilities were available) are omitted.

		**Critical to Semi-Critical**	**Critical to Discharge**	**Critical to Death**
**Pre-Delta period**	**Shape**	1.4548 (1.2483, 1.7096)	19.2673 (10.1777, 40.3650)	0.3469 (0.0476, 0.6481)
	**Scale**	8.7880 (7.7349, 9.8410)	0.5584 (0.3659, 0.7568)	96.7789 (1.6392, 244.4485)
**Delta period**	**Shape**	1.3357 (1.1469, 1.5659)	2.9752 (2.3748, 3.5840)	2.8298 (2.3067, 3.3627)
	**Scale**	9.1888 (7.9565, 10.4275)	3.5919 (3.0222, 4.1754)	4.7125 (4.0544, 5.3714)
**Omicron period**	**Shape**	3.4719 (2.6839, 4.3054)	3.8822 (2.8425, 4.9307)	1.1239 (0.9698, 1.2631)
	**Scale**	1.6738 (1.3771, 1.9679)	1.0985 (0.8977, 1.3032)	3.4728 (2.9323, 4.1013)
**0 – 39**	**Shape**	2.3446 (1.9650, 2.7254)	2.1473 (1.8225, 2.4755)	-
	**Scale**	2.7644 (2.3750, 3.1638)	2.1480 (1.8459, 2.4518)	-
**40 – 64**	**Shape**	1.4483 (1.2981, 1.5981)	5.1124 (3.5373, 6.9499)	1.1458 (0.9117, 1.7507)
	**Scale**	5.7448 (5.0243, 6.4688)	0.9680 (0.7533, 1.1769)	6.4491 (1.4966, 8.0761)
**65 +**	**Shape**	2.4025 (1.9957, 2.8029)	2.3513 (1.9704, 2.7317)	0.6972 (0.2818, 1.1600)
	**Scale**	2.7576 (2.3540, 3.1667)	2.4915 (2.1402, 2.8539)	9.5634 (1.6134, 15.8195)

Table S3-3.

Gamma Fitting Accuracy Metrics (from Semi-Critical ward). RMSE and MAE values computed between the gamma fitting results and the actual data distributions for transitions from the Semi-Critical to the Critical ward, to discharge, or death, across six groups (Pre-Delta, Delta, Omicron, 0–39, 40–64, 65+). These metrics provide a quantitative evaluation of the estimation's performance in capturing the underlying distribution of the observed data.

		Semi-Critical to Critical	Semi-Critical to Discharge	Semi-Critical to Death
Pre-Delta period	RMSE	0.1222	0.0262	0.0577
	MAE	0.1126	0.0214	0.0379
Delta period	RMSE	0.0201	0.0235	0.0326
	MAE	0.0075	0.0174	0.0222
Omicron period	RMSE	0.0076	0.0123	0.0187
	MAE	0.0026	0.0059	0.0153
0 – 39	RMSE	0.0285	0.0171	0.0650
	MAE	0.0194	0.0149	0.0290
40 – 64	RMSE	0.0081	0.0083	0.0392
	MAE	0.0014	0.0037	0.0365
65 +	RMSE	0.0151	0.0154	0.0209
	MAE	0.0086	0.0126	0.0177

Table S3-4.

Gamma Fitting Accuracy Metrics (from Critical ward). RMSE and MAE values computed between the gamma fitting results and the actual data distributions for transitions from the Critical to the Semi-Critical ward, to discharge, or death, across six groups (Pre-Delta, Delta, Omicron, 0–39, 40–64, 65+). These metrics provide a quantitative evaluation of the estimation's performance in capturing the underlying distribution of the observed data.

		Critical to Semi-Critical	Critical to Discharge	Critical to Death
**Pre-Delta period**	**RMSE**	0.0334	0.0552	0.1775
	**MAE**	0.0269	0.0205	0.1609
**Delta period**	**RMSE**	0.0173	0.0350	0.0522
	**MAE**	0.0119	0.0174	0.0291
**Omicron period**	**RMSE**	0.0109	0.0078	0.0076
	**MAE**	0.0025	0.0011	0.0017
**0 – 39**	**RMSE**	0.0422	0.0377	-
	**MAE**	0.0387	0.0256	-
**40 – 64**	**RMSE**	0.0122	0.0092	0.0186
	**MAE**	0.0041	0.0013	0.0048
**65 +**	**RMSE**	0.0201	0.0150	0.0144
	**MAE**	0.0074	0.0042	0.0055
