# Supplementary material for: Multiscale Modeling of Hospital Length of Stay for Successive SARS-CoV-2 Variants: A Multi-State Forecasting Framework
Source: Viruses. 2025 Jul 6;17(7):953. doi: 10.3390/v17070953 (PMC12299293; doi:10.3390/v17070953)

Supplementary S4. Model Estimation Result

For each state transition k, parameters α_k and β_k incorporate the effects of the infection wave (w) and patient age (a) through the following log-linear model:

$$\log(\alpha_k(w,a)) = \mu_{\alpha_k} + \gamma_{\alpha_k}(w) + \delta_{\alpha_k}(a)$$

$$\log(\beta_k(w,a)) = \mu_{\beta_k} + \gamma_{\beta_k}(w) + \delta_{\beta_k}(a)$$

In this equation, $\mu_{\alpha_k}$and μ_{β_k} represent the fundamental shape parameter and scale parameter for transition k, $\gamma_{\alpha_k}(w)$ and $\gamma_{\beta_k}(w)$ represent the effect of the infection wave, and $\delta_{\alpha_k}(a)$, $\delta_{\beta_k}(a)$ capture the effect of age. That is, in this model, the parameters α_k and β_k of the gamma distribution are log-linearly scaled by w and a. Estimated each parameters are shown in Table C1 and Tabel C2.

Table S4-1.

Estimated Model Parameters. (from Semi-Critical ward). This table summarizes the parameters estimated by the model, providing key values and associated statistics that characterize the model's dynamics.

		**Semi-Critical to Critical**	**Semi-Critical to Discharge**	**Semi-Critical to Death**
μ_{α_k}	-	-0.7839	-0.0615	-0.2079
$\gamma_{\alpha_k}(w)$	**Pre-Delta**	0.3447	0.8249	0.7087
	**Delta**	0.1537	0.6200	0.6067
	**Omicron**	0.0591	-0.1649	-0.1820
$\delta_{\alpha_k}(a)$	**0 – 39**	0.3067	0.6667	0.2051
	**40 – 64**	0.0113	0.2221	0.4883
	**65 +**	0.2395	0.3910	0.4399
μ_{β_k}	-	-4.0181	-0.5567	-2.1859
$\gamma_{\beta_k}(w)$	**Pre-Delta**	-1.4386	-0.2949	-0.8227
	**Delta**	-1.3554	-0.4683	-0.6031
	**Omicron**	-2.5656	-1.1351	-2.1016
$\delta_{\beta_k}(a)$	**0 – 39**	-0.7810	0.1382	-2.8554
	**40 – 64**	-3.0994	-0.9737	-0.5494
	**65 +**	-1.4790	-1.0627	-0.1225

Table S4-2.

Estimated Model Parameters. (from Critical ward). This table summarizes the parameters estimated by the model, providing key values and associated statistics that characterize the model's dynamics.

		Critical to Semi-Critical	Critical to Discharge	Critical to Death
μ_{α_k}	-	-0.1176	-0.1087	-0.4237
$\gamma_{\alpha_k}(w)$	**Pre-Delta**	0.2365	0.5937	-0.0491
	**Delta**	0.2991	0.3168	0.7043
	**Omicron**	0.6882	0.3220	0.2624
$\delta_{\alpha_k}(a)$	**0 – 39**	0.3744	0.5769	0.0552
	**40 – 64**	0.2966	0.3703	0.4446
	**65 +**	0.5529	0.2855	0.4178
μ_{β_k}	-	-0.6018	-0.9958	-2.1339
$\gamma_{\beta_k}(w)$	**Pre-Delta**	-1.0428	-0.9496	-2.5729
	**Delta**	-1.0967	-1.1450	-0.3336
	**Omicron**	0.1963	-0.2427	-0.5688
$\delta_{\beta_k}(a)$	**0 – 39**	-0.6299	-0.0195	-2.8522
	**40 – 64**	-0.7964	-1.1169	-0.3990
	**65 +**	-0.5168	-1.2009	-0.2241

Table S4-3.

Model Estimation of Hospital Stay Durations. Estimated mean overall hospital stay duration along with the estimated mean durations for stays in the Semi-Critical and Critical wards, respectively.

		Semi-Critical	Critical	Total
Pre-Delta period	0 – 39	8.5184 (7.6633, 9.3616)	11.2958 (3.0502, 22.7304)	9.6603 (7.9452, 17.2473)
	40 - 64	16.3620 (14.3332, 18.3661)	14.9526 (2.8884, 32.7290)	18.4635 (14.8089, 28.6715)
	65 +	19.9138 (17.8416, 22.1690)	14.3579 (5.9448, 26.8099)	22.3379 (18.4944, 31.3762)
Delta period	0 – 39	8.1840 (7.3173, 9.1405)	11.7752 (5.7657, 19.8189)	9.8252 (7.8696, 17.0063)
	40 - 64	15.2394 (13.1404, 17.3550)	14.0936 (5.8577, 25.8075)	17.32065 (13.9748, 24.6795)
	65 +	17.5619 (15.1963, 20.1671)	13.3257 (7.2708, 20.1742)	19.6897 (16.3201, 25.2107)
Omicron period	0 – 39	7.2127 (6.0505, 8.4905)	4.9678 (1.9724, 10.2444)	7.5205 (6.2879, 9.0603)
	40 - 64	13.0001 (10.4161, 15.7731)	6.1586 (1.7328, 11.6345)	13.4643 (10.8236, 16.3524)
	65 +	15.4481 (12.4219, 18.3792)	5.9807 (2.4262, 10.4752)	15.6272 (12.7417, 18.5235)

Figure S4-4.

Model Simulation Histograms of Age-specific Hospital Stay Durations. Histogram distribution of simulation results segmented by age group for each epidemic period. The simulation was repeated 1000 times to derive average outcomes, and the displayed histogram is one representative example. In the Delta period, which is associated with higher severity, the older age group exhibits a wider distribution extending to longer durations, whereas in the Omicron period, characterized by lower severity, the distributions across age groups are similar.

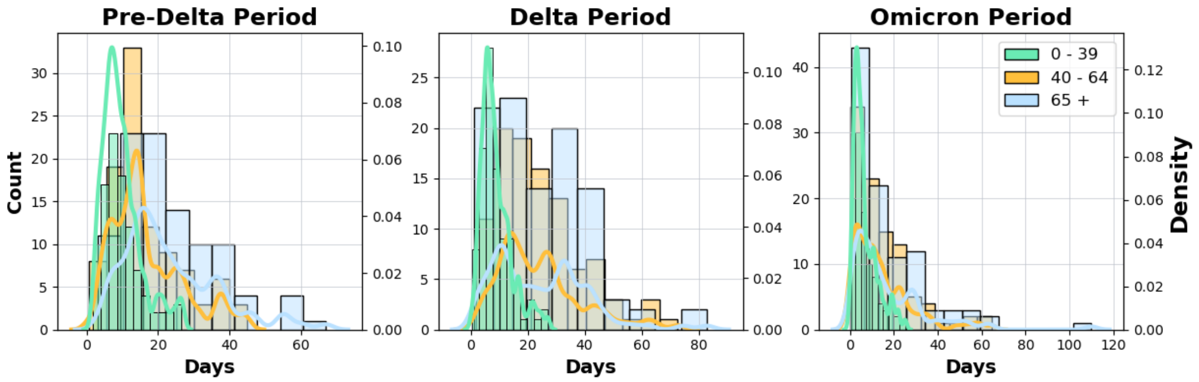

Supplement: Supplementary file 1 [file viruses-17-00953-s001.zip › Supplementary_file_S4.pdf]
